# Bacterial Flora on Mist Outlet Surfaces in 4D Theaters and Suspended Particle Concentration Characteristics during 4D Movie Screenings

**DOI:** 10.3390/microorganisms11071856

**Published:** 2023-07-22

**Authors:** U Yanagi, Noriko Kaihara, Dai Simazaki, Kanae Bekki, Yoshinori Homma, Chiemi Iba, Atsuto Asai, Motoya Hayashi

**Affiliations:** 1 School of Architecture, Kogakuin University, Tokyo 163-8677, Japan; 2 Department of Environmental Health, National Institute of Public Health, Wako 351-0197, Japan; kaihara.n.aa@niph.go.jp (N.K.); simazaki.d.aa@niph.go.jp (D.S.); bekki.k.aa@niph.go.jp (K.B.); honma.y.aa@niph.go.jp (Y.H.); 3 Graduate School of Engineering, Kyoto University, Kyoto 615-8540, Japan; iba@archi.kyoto-u.ac.jp; 4 Graduate School of Engineering, Kogakuin University, Tokyo 163-8677, Japan; dm23003@g.kogakuin.jp; 5 Faculty of Engineering, Hokkaido University, Sapporo 060-8628, Japan; motoya.hayashi@eng.hokudai.ac.jp

**Keywords:** 4D movie theaters, suspended particles, adherent bacteria, mist outlet, α-diversity, β-diversity, next-generation sequencing, 16S rRNA gene

## Abstract

In this study, we measured suspended particle concentrations during the screening of 4D movies (3 screens and 15 movies) and 2D movies (9 screens and 9 movies) in 3 movie theaters to obtain a more detailed understanding of the situation of suspended particle concentrations and adherent bacterial flora in 4D movie theaters, which have been introduced in increasing numbers in recent years. The adherent bacterial flora on the floor and mist outlet surfaces in the 4D movie theaters were collected and analyzed. During the movie showings, the concentrations of suspended particles in 4D movie theaters were significantly higher than those in 2D movie theaters (*p* < 0.001). A significant increase in suspended particle concentrations due to 4D movie effects was also observed. The results of the α-diversity and β-diversity analyses indicate that the bacterial flora on the surfaces of mist outlets in 4D movie theaters are similar. Moreover, there are many closely related species, and the bacterial flora are rich and contain rare bacterial species. Many of the bacterial genera that are dominant in 4D theaters are suited to aqueous environments, and bacteria in the water supply system may have an impact on the indoor environment.

## 1. Introduction

In 2009, the Korean CJ Group company CJ 4Dplex introduced 4DX into its theaters in Korea [1]. As of September 2019, CJ 4DPlex operates 678 4DX theaters in 65 countries via partnerships with more than 80 theaters [2]. By using various effects in 4D movies, viewers can move from “viewing” to “experiencing”. According to the results of Lee et al.’s survey on the frequency of various effects, the order of the frequent effects was motion (58.4%), vibration (21.6%), wind (6.5%), air shots (front) (5.0%), air shots (side) (3.0%), back sweeper (2.8%), water shots (1.3%), strobe light (0.7%), scent, fog, and leg sweeper (0.7%) [3].

So far, research has been conducted on the technical aspects of 4D movie production effects, such as materials [4], satisfaction with the motion effects [5], the effect of effects [6], and the methods of generating 4D effects [7]. However, 4D movie effects include wind, air shots, water shots, fog, fragrance, etc., and these effects may affect indoor air quality (suspended particles and microorganisms). In other words, the concentration of suspended particles in a theater may increase depending on the type of effect, and if the water quality and equipment for water shots are not controlled properly, bacteria may multiply and affect the health of the viewers. We conducted a survey of the literature on suspended particles and microorganisms in 4D movie theaters by using the major databases Scopus, Google Scholar, and PubMed. As far as we have been able to find in the literature, there are no reports of studies on suspended particles and microorganisms in 4D movie theaters.

Many investigations of bacteria in the built environment have been published. The sources of bacteria are also diverse [8], entering from the outside air [9] or originating from occupants [9,10,11,12,13,14,15]. In addition, bacteria and other organisms of the genera *Pseudomonas* and *Methylobacterium* that form biofilms have been detected in heat exchange coils [16,17] and humidifiers [18,19,20] in air conditioning systems in humid environments. However, as mentioned above, no studies on microorganisms related to 4D movie theaters have been found. Therefore, in this study, we measured suspended particle concentrations during movie showings in 4D and conventional 2D movie theaters, collected floor surface-adhering bacteria in both 4D and 2D theaters and mist outlet-adherent bacteria in 4D movie theaters, and analyzed their bacterial flora to understand the situation of indoor suspended particle concentrations and microbial contamination in 4D movie theaters. To the best of our knowledge, this is the first report on the investigation of suspended particle concentrations during movie showings and adherent bacterial flora in 4D movie theaters.

## 2. Materials and Methods

### 2.1. Measured Locations

Measurements were taken in November 2022 in three movie theaters located in Tokyo (movie theater D) and its suburbs (movie theaters C and E). The air-conditioning system in each movie theater is a central system, with a medium-performance air filter installed in the air-handling unit. The air outlet of the air-conditioning system in each movie theater is located on the ceiling, and the air inlet is located at the bottom of the forward screen, so the overall airflow in the movie theater reaches the occupied area from the ceiling and then flows toward the forward screen.

The measurement dates for theaters C, D, and E were 21 November, 25 November, and 29 November 2022, respectively. Measurements were taken in one 4D screen and three 2D screens (all different screens) in each of the movie theaters C, D, and E. For the 4D screens, measurements were taken from the morning screening 1 to the midnight screening 5, and for the 2D screens, measurements were taken during one performance period each. In addition, effects (water shot, air shot, wind, fragrance, fog, and mist) that might affect the indoor air quality were recorded at 5 min intervals during 4D movie showings.

### 2.2. Suspended Particle

Suspended particle concentrations were measured during screenings 1 to 5 in 4D movie theaters and during one screening each (three screenings in total) in 2D movie theaters. Suspended particles were measured continuously at 1 min intervals using particle counters based on the light scattering principle (P611, Airy Technology Japan Ltd., Tokyo, Japan). The particle counters were calibrated by the manufacturer. To ensure the reliability of the results, an instrument difference calibration was performed for the four particle counters beforehand (on the same day, two were used for 4D screens and the other two for 2D screens). The correction coefficients were 0.98–1.03 by particle size. The particle size measurement range of P611 had 6 levels: ≥0.3 μm, ≥0.5 μm, ≥0.7 μm, ≥1.0 μm, ≥2.0 μm, and ≥5.0 μm. The measurement points were placed directly under the seats in the front and rear rows.

### 2.3. Adherent Bacteria

#### 2.3.1. Sampling Method

We had planned to measure suspended bacteria during the screenings, but we were not permitted to do so due to the loudness of the instrument. Therefore, in this study, bacteria on floor surfaces, which represent the history of indoor bacterial contamination, as well as bacteria on the surfaces of mist outlets were collected. For sampling, bacteria on the floor surfaces of the front row and the rear row were collected with an ST-25 PBS wipe kit (Elmex Ltd., Tokyo, Japan) after the last screening 5 in 4D movie theaters and after each screening in 2D movie theaters. The sampling area was 10 cm × 10 cm, and the sampling point was set directly under the seats to avoid aisles. Bacteria adhering to the surfaces of the mist outlets for the water shots in the front and rear rows were also collected with an ST-25 PBS after the 4D movie screenings in movie theaters D and E.

In addition, the number of viewers was counted and carbon dioxide concentrations were measured at 5 min intervals during movie screenings in movie theaters using a CO_2_ recorder (TR-76Ui, T&D Corporation, Matsumoto, Japan). Furthermore, the ventilation rates were estimated from the numbers of viewers and the carbon dioxide concentration measurements.

#### 2.3.2. DNA Extraction, Amplification, and Sequencing

(1) DNA Extraction

After the samples of adhered bacteria were collected as described above, the cotton swabs were processed with a stomacher (MiniMix 100 P CC Interscience, Tokyo, Japan), 3 mL of DNase-free water was combined with 2 mL of each sample solution, and the DNA was extracted with a Stomacher Biomaster device. The processed samples were then removed from the stomacher bag and placed in 1.5 mL test tubes and centrifuged (KUBO-TA5911, Tokyo, Japan) at 4 °C and 3000× *g* rpm for 30 min to extract the bacteria. DNA was purified using a NucleoSpin^®^Tissue kit (740952, MACHEREY-NAGEL, Düren, Germany) and by mixing the process liquid in a vortex mixer.

(2) DNA Amplification and Sequencing

For each sample, variable region 4 (V4) of the bacterial 16S ribosomal RNA (rRNA) gene was amplified via polymerase chain reaction (PCR) using the primers “ACACTCTTTCCCTACACGACGCTCTTCCGATCT-GTGCCAGCMGCCGCGGTAA (1st_515F)” [21], “GTGACTGGAGTTCAGACGTGTGCTCTTCCGATCTGACTACHVGGGTWTCTAAT (1st_806R)”, “AATGATACGGCGACCACCGAGATCTACACxxxxxxxxACACTCTTTCCCTACACGACGC (2nd forward primer)”, and “CAAGCAGAAGACGGCATACGAGATxxxxxxxxGTGACTGGAGTTCAGACGTGTG (2nd reverse primer)”. DNA amplification and 16S rRNA gene analysis were performed using Illumina NGS, and the collected DNA was outsourced to a commercial laboratory.

(3) DNA Sequencing and Analysis

DNA quality was verified using an Agilent 2200 TapeStation (Santa Clara, CA, USA), and all samples containing nucleic acid concentrations of the quality and quantity required for analysis were analyzed. The generated sequence libraries were combined, and the re-amplified PCR products were purified with AMPure XP beads (with a 1:1 bead–volume ratio) to improve the quality of the sequence libraries. Data were analyzed using QIIME (Ver.1.9.0, Silva 132 Database).

### 2.4. Statistical Analysis Methods

In this study, the Mann–Whitney U test using the statistical software IBM SPSS Statistics Ver 29 was used for the differences in suspended particle concentrations by particle size and the alpha diversity values of bacterial flora between 4D and 2D movie theaters. A Mann–Whitney U test can be applied to compare alpha diversity [22]. The data are presented as the medians and interquartile ranges (IQRs). *p*-values of <0.05 were considered statistically significant. Beta diversity was shown via principal coordinate analysis (PCoA) using the weighted UniFrac distance. A weighted UniFrac distance matrix [23,24,25] was calculated using QIIME 2 to compare the microbiome in each sample.

## 3. Results

### 3.1. Suspended Particle Concentrations

**Figure 1:**
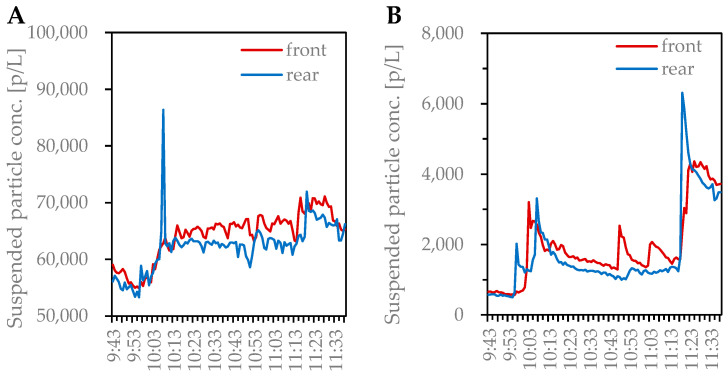
Change over time in suspended particle concentration by particle size (screening 1 in theater E). (**A**): Suspended particle concentration of <1 μm; (**B**): >1 μm suspended particle concentration.

The numbers of effects during the 4D movie screenings in theaters C, D, and E were 218–453, 95–100, and 208–350, respectively (Table S1). As an example of the characteristics of the effects during the 4D movie screenings on the suspended particle concentrations, Figure 1 shows the variation over time in the suspended particle concentrations of <1 μm and >1 μm in the front and rear rows during screening 1 in theater E. Suspended particle concentrations of <1 μm and >1 μm were obtained from measurements of ≥0.3 μm, ≥0.5 μm, ≥0.7 μm, ≥1.0 μm, ≥2.0 μm, and ≥5.0 μm. The movie running time was 9:43–11:38, during which water shot, wind, air shot (front), air shot (foot), fragrance, and fog effects were observed a total of 231 times (Table S1, Figure S1).

For suspended particle concentrations of <1 μm, an increasing trend was observed from 10:00 to 11:20 (Figure 1). Suspended particle concentrations markedly increased at approximately 10:00 (1 water shot, 1 wind shot, 6 air shots (front), and 2 fragrance shots), approximately 10:50 (10 wind shots, 19 air shots (front), 4 air shots (lower part), and 12 fog shot), and approximately 11:20 (3 wind shots, 2 air shots (front), 4 air shots (foot), and 3 fragrance shots), and many effects were observed at those timings (Figure S1). A significant increase in the >1 μm suspended particle concentrations was also observed during these three time periods. The increase in the suspended particle concentrations is thought to be due to the water and fragrance shots, which were then diffused into the theater via air currents. The suspended particle concentrations of <1 μm were 1 order of magnitude higher than those of >1 μm, and the frequency of the rise was also higher than that for >1 μm. On the other hand, a sharp decrease in the suspended particle concentration was observed immediately after an increase (e.g., at approximately 10:10 and 11:20). This was believed to be the effect of dilution due to ventilation. The predicted values of ventilation rates during the movie performance ranged from 27 to 217 m^3^/h/person (Table S2). In general, two–three times the ventilation rate is required to control temperature and humidity; in other words, the actual supply air volume was much higher. The difference in the suspended particle concentrations between the front and rear of the theater is thought to be due to the effect of the airflow distribution in the theater. That is, since the theater was not in a state of instantaneous complete mixing, the concentration differs depending on the location.

**Figure 2:**
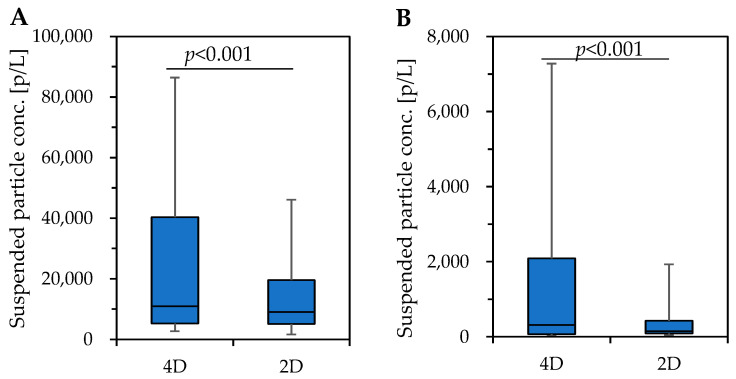
Comparison of suspended particle concentrations during 2D (9 screens) and 4D (15 screens) movie performances. (**A**): Suspended particle concentration of <1 μm; (**B**): >1 μm suspended particle concentration.

For both <1 μm and >1 μm particle sizes, the concentrations in 4D theatres were higher than those in 2D theaters C and E (Figure S2). On the other hand, this trend was not observed in theater D. Incidentally, the number of effects in the 4D screen in theater D was the lowest, averaging at approximately one-third of those in the 4D screens in theaters C and E. Overall, suspended particle concentrations were significantly higher during 4D screenings than during 2D screenings for both <1 μm and >1 μm particle sizes (Figure 2).

### 3.2. Taxonomic Analysis

#### 3.2.1. Taxonomic Identification

Organisms are currently divided into three domains: Eukarya (eukaryotes), Bacteria (eubacteria), and Archaea (archaea). Organisms in each of these domains are further divided by phylum, class, order, family, genus, and species. To allow for comparison with previous reports, this study mainly discusses bacteria in terms of phyla and genera.

#### 3.2.2. Phylum

In total, 17 bacterial phyla were detected. In this section, we describe the bacterial phyla detected at a relative abundance of 1% or higher. *Proteobacteria* (81.70 ± 20.24%), *Actinobacteria* (7.80 ± 20.40%), *Bacteroidetes* (5.30 ± 8.40%), and *Chlamydiae* (1.70 ± 3.92%) were detected in 4D theaters. *Proteobacteria* (71.60 ± 25.44%), *Actinobacteria* (11.22 ± 22.07%), *Bacteroidetes* (8.57 ± 15.76%), *Firmicutes* (2.28 ± 3.57%), and *Cyanobacteria* (0.97 ± 1.77%) were detected in 2D theaters (Figure S3).

#### 3.2.3. Genus

**Figure 3:**
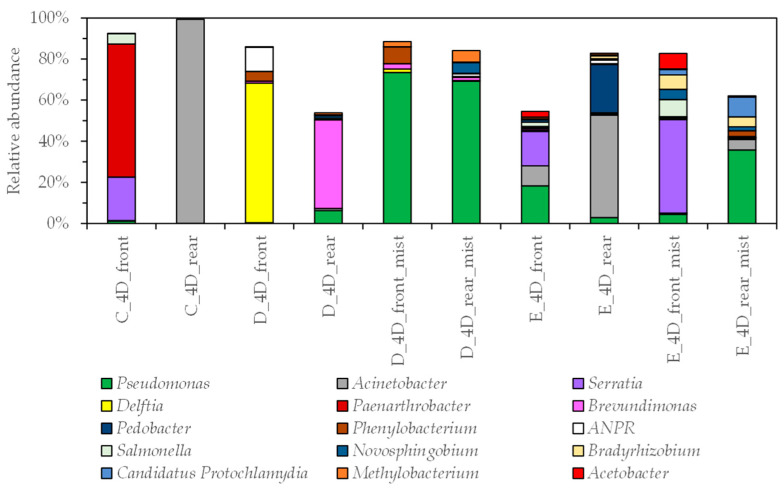
Relative abundance of 1% or higher of bacterial genera for all samples from three 4D theaters. Genus *ANPR*: *Allorhizobium–Neorhizobium–Pararhizobium–Neorhizobim*.

**Figure 4:**
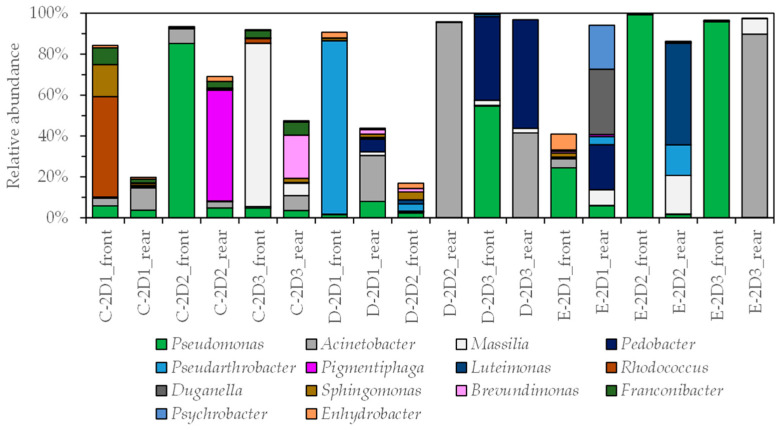
Relative abundance of 1% or higher of bacterial genera for all samples from nine 4D theaters.

A total of four genera, namely *Pseudomonas*, *Acinetobacter*, *Pedobacter*, and *Brevundimonas*, were detected in both 4D and 2D movie theaters at a relative abundance of 1% or higher. Other than the above four genera, the bacterial genera *Serratia*, *Delftia*, *Paenarthrobacter*, *Phenylobacterium*, *Allorhizobium-Neorhizobium-Pararhizobium-Neorhizobium*, *Salmonella*, *Novosphingobium*, *Bradyrhizobium*, *Candidatus Protochlamydia*, and *Methylobacterium* were detected in 4D theaters with a relative abundance of 1% or higher (Figure 3 and Figure 4). *Serratia* are Gram-negative rods belonging to the Enterobacteriaceae family; they are commensal bacteria that are usually found in soil, water, and air. *Serratia marcescens* is a nosocomial pathogen [26]. *Delftia* is a Gram-negative opportunistic pathogenic environmental bacterium [27]. There is a report on the taxonomy of *Paenarthrobacter* [28], but there are few reports of studies in which it is predominantly detected in the built environment. *Phenylobacterium* is a Gram-negative rod bacterium. The first case of cutaneous infectious granuloma caused by this bacterium was reported in 2010 [29]. *Allorhizobium–Neorhizobium–Pararhizobium–Neorhizobium* has been detected and reported in soil [30] and residential settings [17]. *Salmonella* is a Gram-negative bacterium that is widely distributed in nature, including the intestinal tracts of animals such as chickens, pigs, and cattle, as well as rivers and sewage [31]; *Novosphingobium* has been detected in lakes [32]; *Bradyrhizobium* has been detected in soil and water and reported to survive for over one year in distilled water [33]; *Candidatus Protochlamydia* have been detected in groundwater [34]; and *Methylobacterium* is a biofilm-forming bacterium that is frequently detected in bathrooms [35].

For the mist outlets in 4D theaters, *Pseudomonas* were detected on the front mist outlet (D_4D_front_mist) and rear outlet (D_4D_rear_mist) in theater D, and on the front outlet (E_4D_front_mist) and rear outlet (E_4D_rear_mist) in theater E, with a relative abundance of 74%, 69%, 5%, and 36%, respectively. In addition, *P. koreensis*, *P. stutzeri*, and *P. putida* were detected in more than 100 reads for the *Pseudomonas* species. The genus *Methylobacterium* was predominantly detected on the surface of the mist outlet in theater D.

#### 3.2.4. Diversity of Bacterial Community

**Figure 5:**
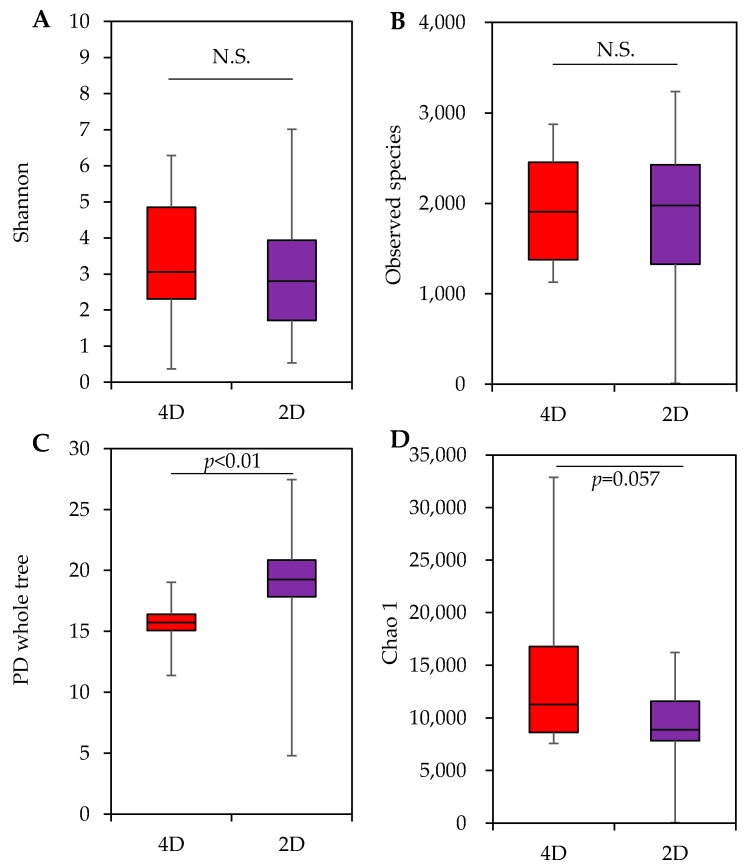
Comparison of the alpha diversity of adherent bacteria between 4D and 2D theaters. (**A**): Shannon Iindex between 4D and 2D theaters; (**B**): Observed species; (**C**): PD whole tree between 4D and 2D theaters; (**D**): Chao 1 between 4D and 2D theaters.

Shannon, Observed species, PD whole tree, and Chao 1 are indexes for quantifying the richness and rarity of the bacteria that make up a bacterial flora. The Shannon index emphasizes the richness of the bacterial species, as well as the bacterial species that are equally detected. The index is higher when the number of bacterial species is high and when each is equally present. The Observed species index counts the abundance of the observed species. The PD whole tree index estimates the abundance of bacteria using phylogenetic distance. PD is calculated as the sum of the lengths of the branches connecting groups of organisms on the phylogenetic tree. The index is smaller if there are more closely related species (closely related species) and larger if there are more distantly related species (distantly related species). The Chao1 index takes into account the abundance of observed species, as well as the rarest of them. Except for the four samples from the mist outlet surfaces in the 4D movie theater, the Mann–Whitney U test showed that the PD whole measurement in the 4D movie theater was lower than that of the 2D movie theater (*p* = 0.047). On the other hand, none of the significant differences between the measurements from the 4D movie theater and 2D movie theater for Shannon, Observed species, and Chao1 were obtained. However, when the 4D movie theater samples were added to the mist samples, the difference in the PD whole index between the 4D movie theater and 2D movie theater was significant (*p* = 0.005), and the Chao 1 index for the 4D movie theater was higher than that for the 2D movie theater (*p* = 0.057) (Figure 5).

**Figure 6:**
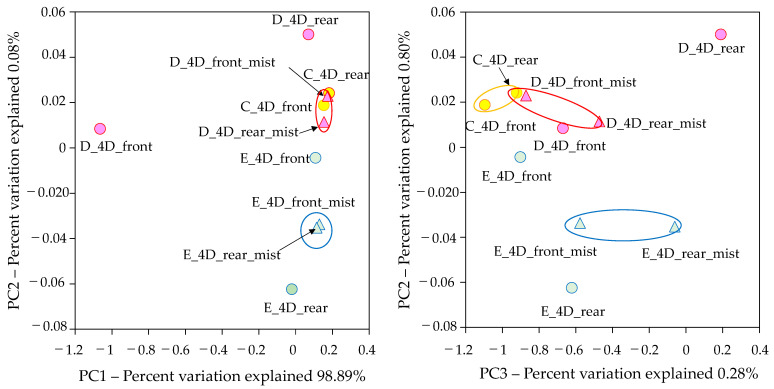
Principal coordinate analysis (PCoA) of the weighted UniFrac distance of 4D theaters.

Beta diversity was shown via principal coordinate analysis (PCoA) using the weighted UniFrac distance. The results of the principal coordinate analysis show that the bacterial flora on the front and rear mist outlet surfaces in the 4D theaters D and E and the floor-adherent bacterial flora in the front and rear of the 4D theater C were similar. On the other hand, the flora in the fronts and rears of theater D and theater E were different (Figure 6).

## 4. Discussion

In this study, we measured suspended particle concentrations during the 4D movie screenings (3 screens and 15 movies) and 2D movies (9 screens and 9 movies) in three movie theaters to obtain a more detailed understanding of the situation of suspended particle concentrations and adherent bacterial flora in 4D movie theaters, which have been introduced in increasing numbers in recent years. The adherent bacterial flora on the floor and mist outlet surfaces in 4D movie theaters were collected and analyzed. Concerning suspended particle concentrations, the <1 μm and >1 μm concentrations were significantly higher during the screening of 4D movies than 2D movies (*p* < 0.001). This can be explained by the significant increase in suspended particle concentrations due to effects during the 4D movie. This may have been because particulate matter emitted from water and fragrance shots contributed to the increase in suspended particle concentrations. In addition, the concentration of particles of <1 μm was 1 order of magnitude higher than that of suspended particles of >1 μm, indicating that most particles emitted via the effects were submicron particles. The impact of airborne particles on human health is related to their particle size and composition. Depending on the size of a particle, its site and deposition efficiency in the respiratory system will differ. It is known that most large particles (>1 μm in size) are deposited in the nasopharyngeal region, while most small particles (<1 μm in size) can reach and be deposited in the bronchial and alveolar region [36,37]. On the other hand, as this study was conducted during movie screenings, sampling to identify the composition of the suspended particles was not possible due to various restrictions. This issue needs to be examined in the future, including the measurement methods. As a countermeasure, dilution via ventilation is effective. In the actual case, the concentrations of suspended particles rapidly increased and then quickly decayed (Figure 1). This result suggested that the ventilation was effective.

As for the phyla of adherent bacteria on the floor, *Proteobacteria*, *Actinobacteria*, *Bacteroidetes*, and *Chlamydiae* were found in 4D movie theaters, and *Proteobacteria*, *Actinobacteria*, *Bacteroidetes*, *Firmicutes*, and *Cyanobacteria* were found in 2D movie theaters with a relative abundance of 1% or higher. The phyla *Proteobacteria*, *Actinobacteria*, *Bacteroidetes*, *Firmicutes*, and *Cyanobacteria*, excluding the *Chlamydiae* phylum, have also been detected predominantly in house dust [13,38], air-conditioning systems [17,39], university laboratory air, the mouths and hands of occupants, doorknobs [15], residential carpets and floors [40], and airplane HEPA filter surfaces [41]. These findings suggest that these bacterial phyla favor growth in the built environment. On the other hand, the phylum *Chlamydiae* detected on the mist outlets of the 4D screen in movie theater E is known to contain pathogenic species of eye infections, respiratory tract infections, and sexually transmitted diseases [42]. To date, few cases of the detection of the *Chlamydia* phylum as the dominant phylum in the built environment have been reported.

For bacterial genera, *Pseudomonas*, *Acinetobacter*, *Brevundimonas*, and *Pedobacter* were commonly detected in both 4D and 2D movie theaters at a relative abundance of 1% or higher. *Brevundimonas* have been detected in retail stores [10], indoor environments [11], classrooms [12], and neonatal intensive care units [43], and *Pedobacter* has been detected in drinking water [44] and soil [45]. *Candidatus Protochlamydia* of the phylum *Chamydiae*, which have not been detected in other locations, were detected on the mist outlets in movie theater E (E_4D_front_mist and E_4D_rear_mist) and the floor in the rear (E_4D_rear), with reads of 2465, 7162, and 725, respectively. Similarly, the genus *Neochlamydia* of the phylum *Chamydiae* was detected on the mist outlet surface (E_4D_front_mist, E_4D_rear_mist) and the rear floor surface (E_4D_rear) in movie theater E with a relative abundance of 0.4% and reads of 799, 2222, and 354, respectively, indicating that bacteria in the mist were dispersed into the movie theater. The above two bacterial species were detected on the floor surface, which is thought to be related to the airflow in the movie theater. That is, as mentioned above, the airflow in a movie theater generally flows from the ceiling toward the screen in front after reaching the occupied area, so some of the bacteria released with the mist fall onto the floor. *Candidatus Protochlamydia* has been found in groundwater [34], the HS-T3 amoeba (Acanthamoeba) was isolated from hot springs [46], and the genus *Neochlamydia* has been detected in drinking water systems [47]. These two genera are bacteria that can live in water. In addition to these two genera, *Serratia*, *Salmonella*, *Novosphingobium*, *Bradyrhizobium*, and *Methylobacterium*, which were detected predominantly in 4D movie theaters but not in 2D movie theaters at a relative abundance of 1% or higher, are bacteria that are suited to aqueous environments; therefore, it is assumed that they are related to the mist supply system.

Regarding α-diversity, except for the four samples from the mist outlet surfaces in 4D movie theaters, the Mann–Whitney U test showed that 4D movie theaters were lower than 2D movie theaters in PD whole (*p* = 0.047). On the other hand, none of the significant differences between 4D movie theaters and 2D movie theaters in Shannon, Observed species, and Chao1 were obtained. However, when the 4D movie theater samples were added to the mist samples, the significant difference in PD whole index between 4D movie theaters and 2D movie theaters were significant (*p* < 0.005), and the Chao 1 index in the 4D movie theaters were higher than those in the 2D movie theaters (*p* = 0.057). The low PD whole tree index means that the bacteria on the surfaces of 4D theater air outlets are mostly bacterial species (closely related species) that are close in position on the phylogenetic tree. As mentioned earlier, many of the bacterial genera detected in the 4D movie theaters with a relative abundance of 1% or higher are suited to aqueous environments, which may have influenced the results. In addition to the richness of the observed bacterial species, Chao1 is an indicator that takes into account the rarest of those species, and it is likely that the adherent bacteria on the surfaces of mist outlets in 4D movie theaters were rich and contained rare species. These changes in the PD whole tree and Chao 1 indexes by adding samples from the surfaces of the mist outlets indicate that the bacteria on the mist outlet surfaces are a closely related species, have a rich flora, and contain rare bacterial species.

Regarding β-diversity, the similarity of the bacterial flora on the mist outlet surfaces in the fronts and rears of movie theater D and movie theater E may be because they originate from the same water supply system. As mentioned earlier, many of the dominant bacterial genera found on the mist outlet surfaces are suited to aqueous environments, suggesting that the bacteria in the water supply system may have an impact on the indoor environment. These results suggest that in indoor environments where water is used, other than 4D movie theaters, sanitation control of water is also important.

As this study did not use the culture method, it was not possible to determine whether the bacteria detected were viable or not. Incidentally, the water supply systems were supplied with public water suppliers, and in the case of Japan, the water should be disinfected with chlorine.

## 5. Conclusions

To the best of our knowledge, this is the first study to measure suspended particle concentrations during 4D movie screenings, floor surface microbiota, and mist outlet surface microbiota in 4D movie theaters. The suspended particle concentrations in 4D movie theaters were significantly higher than in 2D movie theaters (*p* < 0.001). There was also a significant increase in suspended particle concentrations due to 4D movie effects. Regarding suspended particle concentrations by particle size, the concentration of particles of <1 μm was 1 order of magnitude higher than that of particles of >1 μm. Most of the suspended particles generated via the effects were submicron particles. Regarding the adherent bacterial flora, the results of the diversity analysis show that the adherent bacteria on the surfaces of the mist outlets in 4D movie theaters were rich and included some rare species. Most of the dominant bacterial genera found on 4D theaters surfaces are suited to aqueous environments (*Candidatus Protochlamydia*, *Neochlamydia*, *Serratia*, *Salmonella*, *Novosphingobium*, *Bradyrhizobium*, and *Methylobacterium*). Furthermore, bacteria in the water supply system may have an impact on the indoor environment, so sanitizing the water supply system is important.

## Data Availability

Not applicable.

## Supplementary Materials

The following supporting information can be downloaded at: https://www.mdpi.com/article/10.3390/microorganisms11071856/s1. References [48,49,50] are cited in the supplementary materials.
