# Supplementary material for: Bacterial Flora on Mist Outlet Surfaces in 4D Theaters and Suspended Particle Concentration Characteristics during 4D Movie Screenings
Source: Microorganisms. 2023 Jul 22;11(7):1856. doi: 10.3390/microorganisms11071856 (PMC10383669; doi:10.3390/microorganisms11071856)
Supplement: Supplementary file 1 [file microorganisms-11-01856-s001.zip › microorganisms-2503879-supplementary.pdf]

Literature review

We conducted a survey of the literature on suspended particles and microorganisms in 4D movie theaters. The major databases Scopus, Google Scholar, and PubMed were used for the literature review, and the survey covered the period until May 31, 2023. A search using the keyword "4D cinema" yielded 9, 331, and 35 results in Scopus, Google Scholar, and PubMed, respectively, and a search using the keyword "4D movie theater" yielded 7, 81, and 8 hits, respectively. However, for suspended particles, searches for the keywords "4D cinema" AND "suspended particle" and "4D theater" AND "sus-pended particle" returned only 16 hits in Google Scholar. However, not all of the 16 results were related to suspended particles. As for microorganisms, a search for the keywords "4D theater" AND "microorganisms" yielded zero hits, and the keywords "4D cinema" AND "microorganisms" yielded only three hits in Google Scholar. None of the three hits were related to microorganisms in 4D theaters [1-3]. That is, as far as we have been able to find in the literature, there are no reports of studies on suspended particles and microorganisms in 4D movie theaters.

References

1. Haris, D.; Nasirin, Dahlan, D.; Abidin, G.Z.; Partono, C.E.; Budihadi, A. Slurry Ice Machine Design Production Capacity of 1.3 Tons. Int. J. Eng. Res. 2020, 15(3), 212-215.
2. Editing: Hentzsch, B. Science Meets School, Examples from the South Baltic Area. 2013.
3. Firebrace, W. Star theatre: the story of the planetarium. 2017, https://www.reaktionbooks.co.uk

Table S1. Number of effects during the 4D movie screenings in theaters C, D, and E.

		Water shot	Wind	Air shot (front)	Air shot (foot)	Fragrance	Fog	Mist (head)	Total
Theater C	movie 1	2	158	71	53	3	0	0	287
	movie 2	19	96	124	206	4	0	4	453
	movie 3	2	158	71	53	3	0	0	287
	movie 4	14	85	74	38	3	0	4	218
	movie 5	19	96	124	206	4	0	4	453
Theater D	movie 1	3	27	31	29	2	3	0	95
	movie 2	3	27	31	29	2	3	0	95
	movie 3	3	27	31	29	2	3	0	95
	movie 4	4	32	46	15	2	1	0	100
	movie 5	4	32	46	15	2	1	0	100
Theater E	movie 1	8	52	77	69	21	1	3	231
	movie 2	8	52	77	69	21	1	3	231
	movie 3	31	51	160	64	2	0	0	308
	movie 4	36	61	173	77	3	0	0	350
	movie 5	19	42	100	42	5	0	0	208

Table S2. Number of viewers, CO_2 concentrations, and predicted ventilation rates during the movie screening periods.

		Occupants	CO_2 concentration [ppm]	Ventilation rate [m^3/h/person]
Theater C 4D	movie 1	32	901±164	40
	movie 2	21	738±159	59
	movie 3	35	669±87	74
	movie 4	15	729±91	61
	movie 5	15	702±73	66
Theater C 2D	movie 1	37	769±125	54
	movie 2	75	1151±47	27
	movie 3	24	701±26	66
Theater D 4D	movie 1	13	499±23	202
	movie 2	11	492±36	217
	movie 3	11	557±26	127
	movie 4	14	601±33	100
	movie 5	12	650±31	80
Theater D 2D	movie 1	78	704±61	66
	movie 2	44	730±82	61
	movie 3	20	761±39	55
Theater E 4D	movie 1	11	564±24	122
	movie 2	13	583±24	109
	movie 3	18	675±45	73
	movie 4	13	696±26	68
	movie 5	12	636±24	85
Theater E 2D	movie 1	12	546±26	137
	movie 2	21	555±22	129
	movie 3	21	643±63	82

The ventilation rates were calculated from the ratios of the amount of carbon dioxide generated and the difference between the mean indoor and outdoor carbon dioxide concentrations. Here, it was assumed that the amount of carbon dioxide generated was 20 L/h/person and the concentration of carbon dioxide in the outside air was 400 ppm.
The carbon dioxide concentrations were measured at 5-minute intervals during the movie screening periods using a CO_2 recorder (TR-76Ui, T&D Corporation).

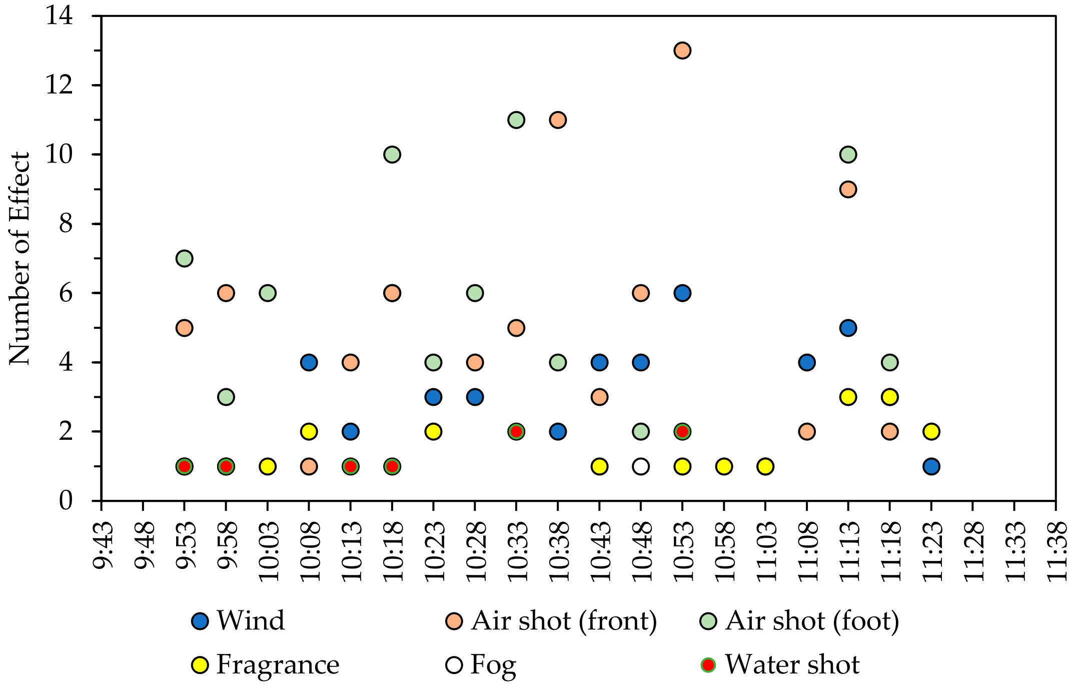

Figure S1. Number of effects by time during screening one in theater E.

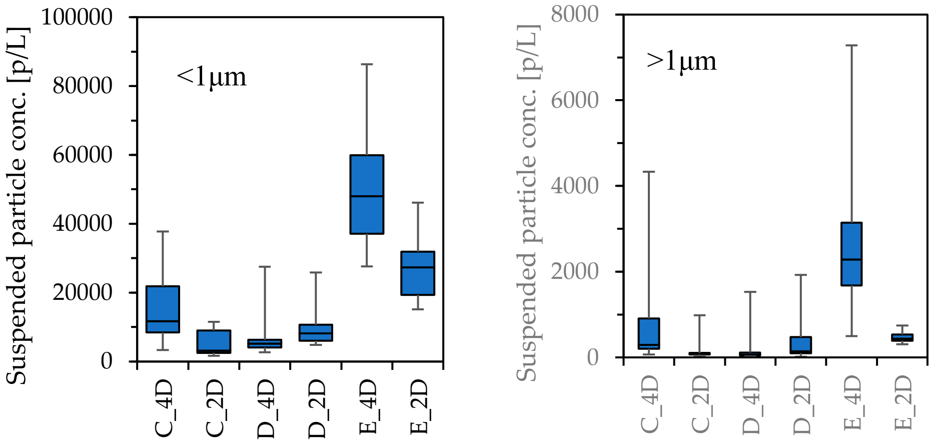

Figure S2. Particle concentrations in the 2D (three movies on different screens) and 4D (five movies on the same screen) theaters during the movie screenings.

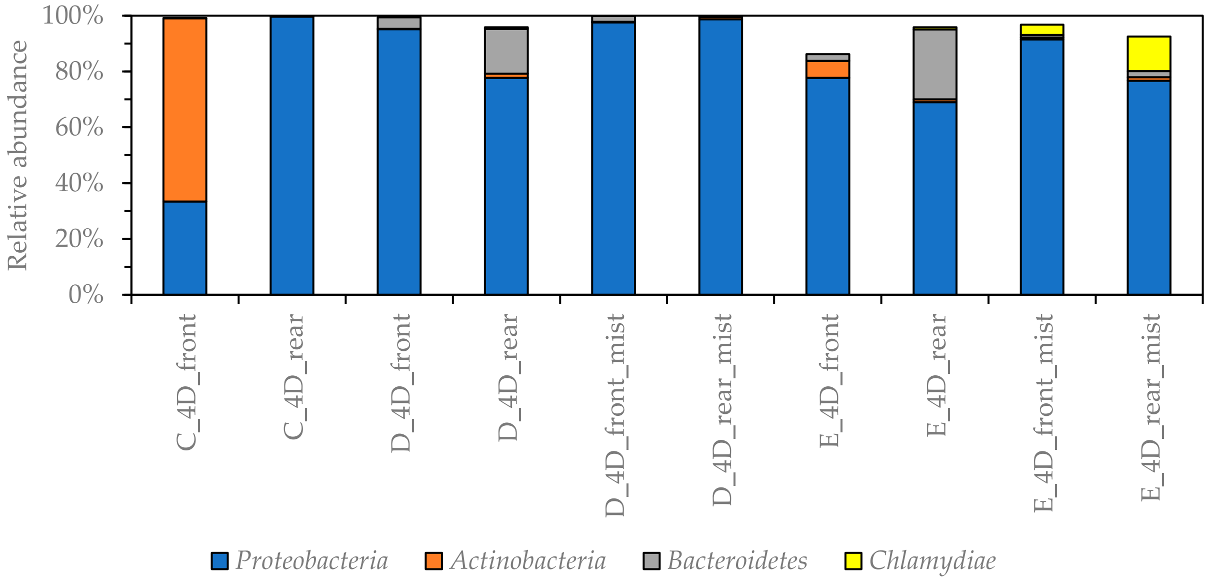

Figure S3. Relative abundance of 1% or higher of bacterial phyla for all samples from the 3 4D movie theaters.

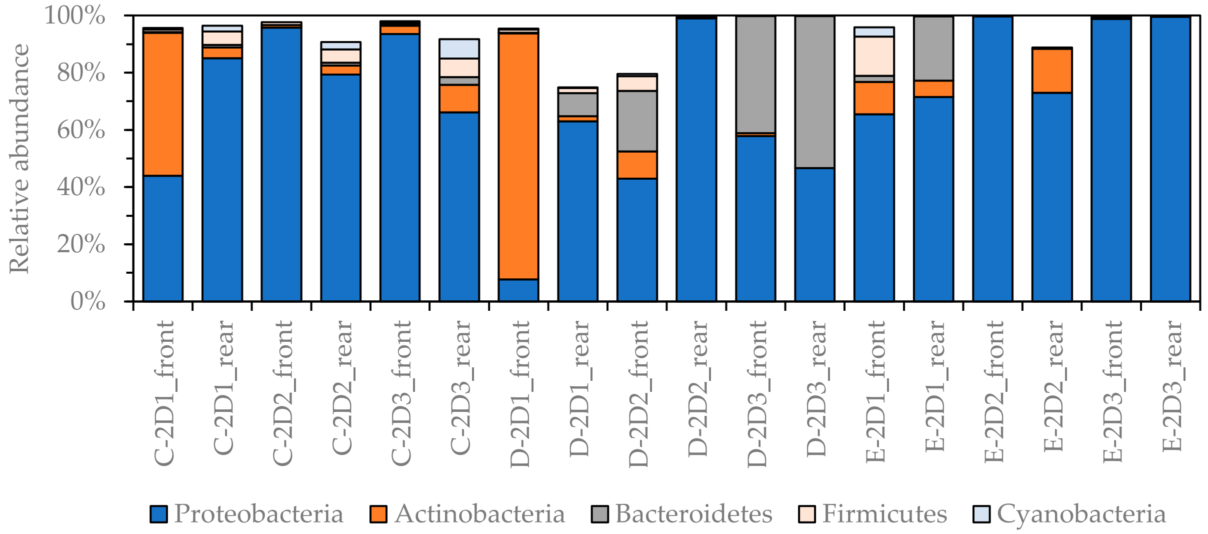

Figure S4. Relative abundance of 1% or higher of bacterial phyla for all samples from the 9 2D movie theaters.
